# The pro-oxidative drug WF-10 inhibits serial killing by primary human cytotoxic T-cells

**DOI:** 10.1038/cddiscovery.2016.57

**Published:** 2016-07-25

**Authors:** G H Wabnitz, E Balta, S Schindler, H Kirchgessner, B Jahraus, S Meuer, Y Samstag

**Affiliations:** 1 Institute of Immunology, Ruprecht-Karls-University, Im Neuenheimer Feld 305, Heidelberg D-69120, Germany

## Abstract

Cytotoxic T-cells (CTLs) play an important role in many immune-mediated inflammatory diseases. Targeting cytotoxicity of CTLs would allow to interfere with immune-mediated tissue destruction. Here we demonstrate that WF-10, a pro-oxidative compound, inhibits CTL-mediated cytotoxicity. WF-10 did not influence early steps of target-cell killing, but impaired the ability of CTLs to detach from the initial target cell and to move to a second target cell. This reduced serial killing was accompanied by stronger enrichment of the adhesion molecule LFA-1 in the cytolytic immune synapse. LFA-1 clustering requires activation of the actin-bundling protein L-plastin and was accordingly diminished in L-plastin knockdown cells. Interestingly, WF-10 likely acts through regulating L-plastin: (I) It induced L-plastin activation through phosphorylation leading to enhanced LFA-1-mediated cell adhesion, and, importantly, (II) WF-10 lost its influence on target-cell killing in L-plastin knockdown cells. Finally, we demonstrate that WF-10 can improve immunosuppression by conventional drugs. Thus, while cyclosporine A alone had no significant effect on cytotoxicity of CTLs, a combination of cyclosporine A and WF-10 blocked target-cell killing synergistically. Together, our findings suggest that WF-10 – either alone or in combination with conventional immunosuppressive drugs – may be efficient to control progression of diseases, in which CTLs are crucially involved.

## Introduction

T-cells are activated in lymph nodes through interaction with antigen-pres0enting cells (APCs). This activation is initiated at the contact zone between T-cells and corresponding APCs, called immune synapse.^1^ We identified regulators of the actin cytoskeleton that are important to initiate and to sustain the immune synapse, that is, cofilin and L-plastin.^2–5^ While the actin-depolymerizing molecule cofilin induces actin dynamics,^6,7^ L-plastin regulates actin bundles and LFA-1 avidity within the immune synapse.^4^ This T-cell intrinsic regulation of the immune synapse is modulated by extrinsic factors of the microenvironment. In this respect, we have shown earlier that oxidative stress, as induced by H_2_O_2_, provokes an immediate inhibition of T-cell activation through a miss-regulation of the actin cytoskeleton and immune synapse formation.^2,8^ Thus, a pro-oxidative microenvironment has the potential to switch the behavior of immunocompetent cells from an inflammatory into a tolerogenic or anergic state by regulating the functional plasticity of T-cells.

After their initial activation, CD8^+^ T-cells become armed effector cells (cytotoxic T lymphocytes or CTLs). CTLs migrate into inflamed tissues and kill tumor cells or virus-infected cells. During this distal effector phase, CTLs form short-lived immune synapses with their target cells, called cytolytic immune synapses. CTLs secrete lytic granules into the cytolytic immune synapse in a calcium-dependent manner.^9^ This polarized degranulation is important for effective target cell lysis and it protects innocent bystander cells (reviewed in Reichardt *et al*.^10^). However, cytotoxicity of T-cells also needs to be tightly controlled, since inappropriate cytotoxicity can lead to chronic inflammatory diseases and tissue destruction.^11–15^ Therefore, it is important to understand the basal mechanisms of T-cell cytotoxicity and to find means to prevent self-directed cytotoxicity of T-cells. In that regard, the effects of micromilieu-modifying agents on the function of CTLs and the cytolytic immune synapse are as yet largely unknown. One main question was, therefore, if and how a therapeutic manipulation of the redox balance can be achieved to control the functional plasticity within tissues and whether such a treatment would influence the T-cell effector phase. WF-10 (Immunokine) is a chlorite-containing compound that is stable in aqueous solution.^16,17^ It generates oxidative species such as taurine-chloramine and mono-chloramine in the cytoplasm of cells.^16^ It is known to inhibit proliferation and IL-2 production of CD3-stimulated PBMC,^18^ while innate immunity by macrophages and NK cells was rather enhanced.^19,20^ On the contrary to other reactive oxygen species (ROS), for example, H_2_O_2_, WF-10 can be intravenously injected. It was initially developed to control wound healing and opportunistic infections during acquired immunodeficiency syndrome.^21^ Clinically, it is also administered during chronic inflammatory disorders, as, for example, proctitis, cystitis or mucositis.^16,22,23^

In the current study, we discovered that WF-10 inhibits serial killing of target cells by CTLs. This inhibition was associated with an increased localization of LFA-1 in the cytolytic immune synapse, an elevated level of F-actin and a hyper-phosphorylation of L-plastin. Interestingly, the clinically used immunosuppressive drug CsA did not reduce CTL-mediated killing. However, a combination of CsA with WF-10 strongly amplified the inhibitory capacity of WF-10. Together, our data suggest that WF-10 may be a tool to control disease progression in which tissue destruction by cytotoxic T-cells occurs.

## Results

### WF-10 downregulates cytotoxicity of human CD8+ T-cells

**Figure 1:**
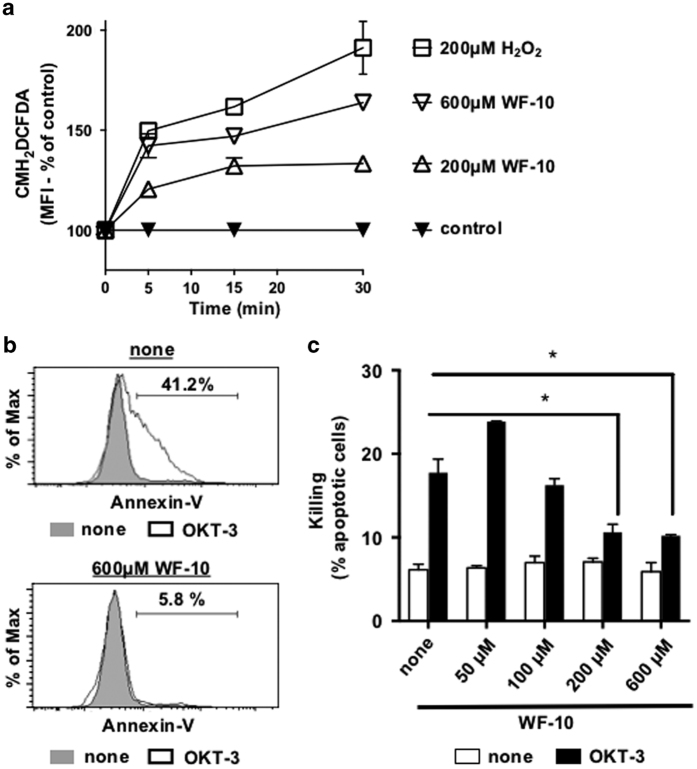
WF-10 inhibits CTL-mediated target cell killing in a dose-dependent manner. (**a**) To assess ROS level, human CTLs were labeled with the ROS sensor CM-H_2_DCHDA and incubated with 600 or 200 *μ*M WF-10 or 200 *μ*M H_2_O_2_. ROS content was measured using flow cytometry. Shown is the relative increase of the MFI of CMH_2_DCFDA (*n*=2; S.E.M.). (**b**) CTLs were preincubated with 600 *μ*M WF-10 (lower panel) or solvent control (upper panel) for 2 h. Thereafter, T-cells were mixed with target cells (P815) that were either coupled to OKT3 (OKT-3) or not (none). Target cell death was analyzed via Annexin-V binding and analyzed using flow cytometry. Percent Annexin-V positive indicates apoptotic target cells. (**c**) The graph shows a titration of WF-10 in a redirected lysis assay as described in (**b**) (*n*=3; S.E.M.; *P<0.05).

We have recently shown that a pro-oxidative milleu influences the actin-reorganizing protein cofilin, thereby interfering with activation of naïve human T-cells.^8,24^^,^^25^ WF-10 is a compound that is stable in aqueous solution and is converted to chloramins in cellular environments (Supplementary Figure S1). As shown in Figure 1a, exposure of primary human CTLs (PHA/IL-2 stimulated primary human CD8^+^ T-cells) to WF-10 leads to pro-oxidative intracellular conditions. We therefore reasoned that WF-10 could influence actin-dependent functions of CTLs, that is, cytotoxicity, in the distal phase of adaptive immune responses. To investigate that point, we used the mastocytoma cell line P815 as a target cell. P815 carry Fc receptors enabling the loading of monoclonal antibodies for CTL stimulation. CTL-mediated targeted cell killing was induced by adding anti-CD3 antibodies (OKT-3) and analyzed by Annexin-V staining and flow cytometry (Figure 1b, upper part). The amount of target cell killing via CTLs varied between blood donors from 20 to 80% apoptotic target cells. Independent of this donor-specific variations, we found that preincubation of CTLs with 600 *μ*M WF-10 for 30 min almost completely inhibited CTL-mediated killing (Figure 1b, lower part). Note that, preincubation of the target cells with WF-10 alone had no effect on target cell death (not shown). Next, we performed a titration of WF-10 from 50 to 600 *μ*M (Figure 1c). A significant reduction of CTL-mediated killing could be observed from 200 *μ*M WF-10 and above, which are considered therapeutic concentrations.^20,22^ To scrutinize whether a similar result could be achieved with another pro-oxidative reagent, namely H_2_O_2_, we incubated CTLs with 50 *μ*M of H_2_O_2_ prior to the redirected killing assay (Supplementary Figure S2A). For comparison, we treated the cells with the reducing agent *N*-acetylcysteine (NAC). As observed for WF-10 the ability of CTLs to kill their target cells was inhibited, when a strong pro-oxidative environment was produced by H_2_O_2_. In opposite, a change in the redox-milieu towards reducing conditions by NAC did not influence target cell killing. Thus, pro-oxidative conditions interfere with cytotoxic effector functions of CTLs.

### Intact granzyme polarization and degranulation of WF-10-treated CTLs

**Figure 2:**
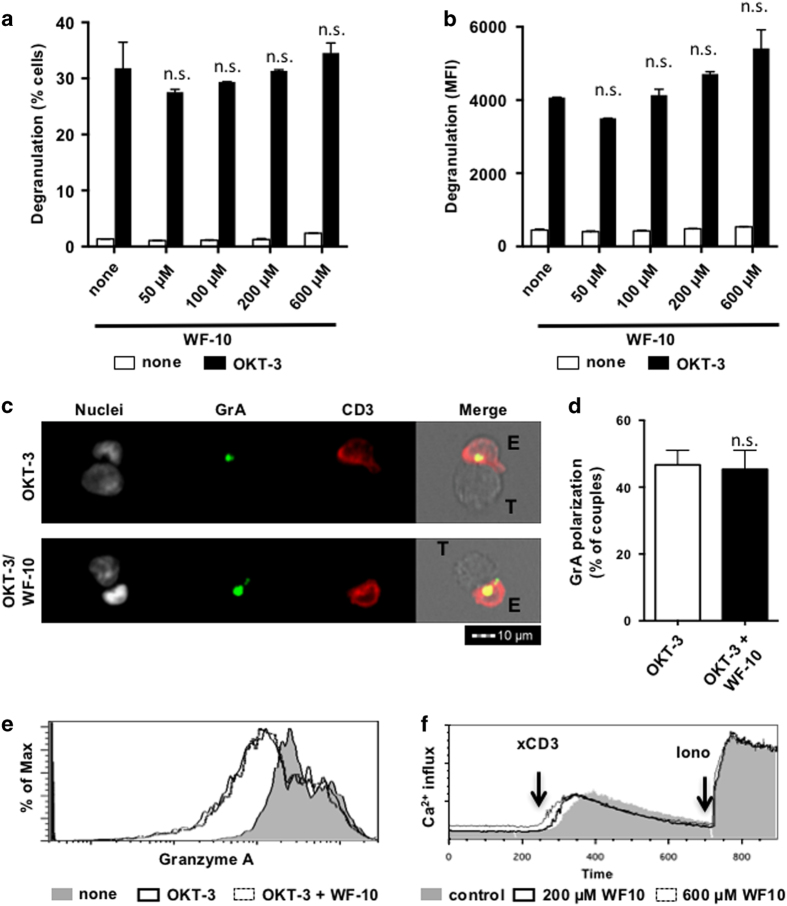
Intact degranulation, granzyme A polarization and calcium mobilization by WF-10-treated CTLs. (**a**, **b**) CTLs were preincubated with WF-10 as indicated and the redirected killing assay was performed in the presence of APC-labeled CD107a-specific antibodies. Degranulation was quantified by measuring anti-CD107a antibody binding using flow cytometry. Shown are percent CD107a-positive CTLs (**a**) and the MFI (**b**) of CD107a. (**c**, **d**) The localization of granzyme A (GrA) was analyzed using InFlow microscopy. Depicted are representative images of E/T couples without (**c**, upper panel) or with (**c**, lower panel) WF-10 (200 *μ*M) preincubation of CTLs. A quantification of the number of E/T couples, in which GrA was polarized to the cytolytic immune synapse, is shown in (**d**) (*n*=3; S.E.M.; NS= not significant). (**e**) GrA expression was measured in CTLs that were mixed with target cells that were unloaded (none) or loaded with OKT3 using InFlow microscopy. In the latter case, CTLs were either pretreated with WF-10 (200 *μ*M) or kept untreated. The histogram is representative for three independent experiments. (**f**) Calcium influx in CTLs that were either treated with solvent control, 200 or 600 *μ*M WF-10 was measured using flow cytometry. Cells were stimulated by crosslinking CD3 as indicated. Ionomycin was added as a positive control at the end of each measurement. The depicted graph is representative for three independent experiments.

To gain insight into mechanisms for the observed inhibition of CTL-mediated target cell killing by WF-10, we analyzed functionally important events during CTL-mediated killing, that is, degranulation, granzyme A (GrA) polarization and calcium influx. Degranulation was measured by surface expression of CD107a using flow cytometry.^26^ CD107a is a component of the membrane of lytic vesicles and is transported to the cell surface during degranulation. To trap CD107a a fluorescent-labeled antibody against CD107a was added during the killing assay. Stimulation of CTLs with OKT-3-bearing P815 induced an increase of degranulating cells from 1.4±0.2% (without OKT-3) to 30.9±1.1% (with OKT-3) (Figure 2a) and a rise of the MFI from 457±23 (without OKT-3) to 4367±324 (with OKT-3) (Figure 2b). Although WF-10 inhibited CTL-mediated killing in a dose-dependent manner (compare Figure 1), there was no reduction in the percentage of CD107a-positive cells or the MFI of CD107a if CTLs were preincubated with WF-10 (Figures 2a and b). Similarly, H_2_O_2_ did not reduce the amount of CD107a-positive cells (Supplementary Figure S2B). Thus, although CTL-mediated killing was reduced, CTLs were still able to degranulate under pro-oxidative conditions.

Granzymes and perforines need to be secreted into the immune synapse for an efficient induction of target cell death. We therefore scrutinized whether GrA polarization towards the target cells was affected by WF-10. To this end, we took advantage of InFlow microscopy, which is a combination of flow cytometry and fluorescence microscopy.^27^ We acquired images of 15 000 cells in total, which ends up in about 200 effector/target (E/T) couples that could be analyzed. Using this method, we found that WF-10-treated CTLs were able to polarize GrA into the cytolytic immune synapse to the same degree as observed for control cells (OKT-3) (Figures 2c and d). Moreover, the amount of intracellular GrA in CTLs decreased similarly in untreated or WF-10 pretreated CTLs (Figure 2e). This confirms our previous finding that degranulation is unaffected by WF-10.

To obtain information on the activities of granzymes and perforines, we analyzed calcium mobility, since degranulation and perforine polymerization are calcium-dependent processes.^28^ Calcium influx was analyzed by crosslinking of cell-bound CD3 antibodies followed by life-cell flow cytometry. A calcium influx could be equally induced in cells that were either treated with 200 *μ*M WF-10, 600 *μ*M WF-10 or solvent control (Figure 2f). Together, the killing machinery, that is, granule expression, polarization, degranulation and activation, appears not to be inhibited by WF-10.

### WF-10 interfered with serial killing

**Figure 3:**
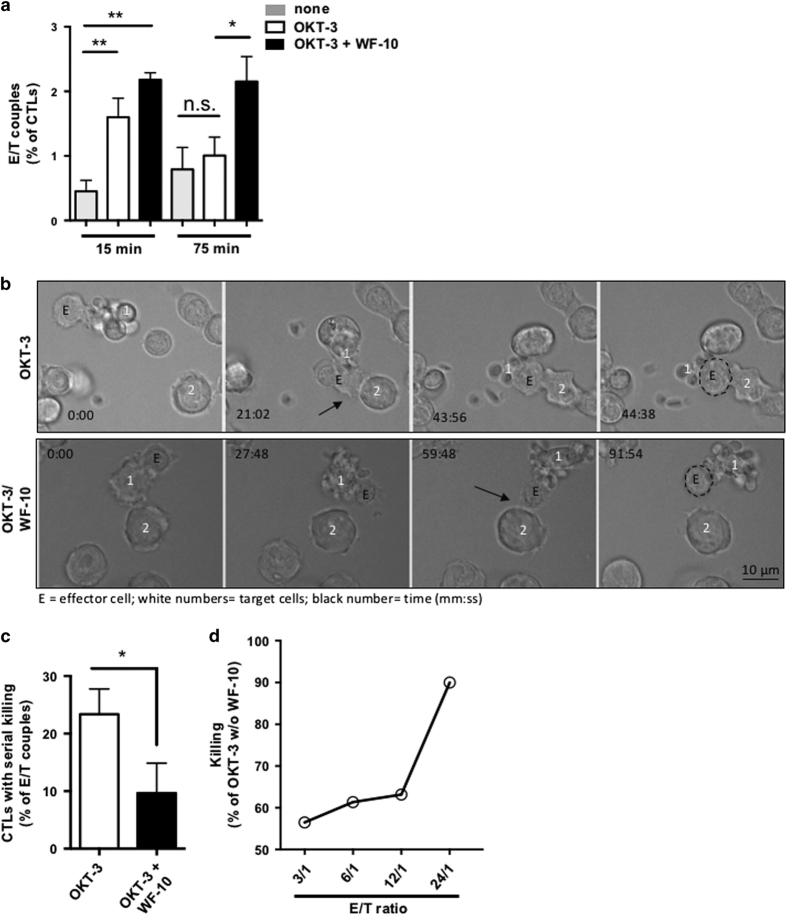
WF-10 disturbed serial target cell killing by human CTLs. (**a**) E/T couple formation was analyzed after 15 or 75 min as indicated using InFlow microscopy. Shown is the percentage of CTLs that formed couples with target cells that were either unloaded or loaded with OKT-3. CTLs were either preincubated with WF-10 (200 *μ*M) or not (*n*=3; S.E.M.; *P<0.05; **P<0.01, NS=not significant). (**b**) Images from redirected killing assay using OKT-3-loaded target cells. CTLs were either preincubated with WF-10 (200 *μ*M) (lower panel) or not (upper panel). The time (black numbers) indicates minutes and seconds (min:s). Target cells are labeled with white numbers. The black arrows mark the first contact with the second target cell. The border of the effector cell is highlighted in the last image by a dotted line, respectively. Images are representative for three independent experiments, in which up to 50 E/T couples were evaluated. (**c**) Quantification of CTLs that performed serial killing in the absence (white) or presence (black) of WF-10 (200 *μ*M). The numbers show the percentage of CTLs that formed at least two consecutive E/T couples (*n*=3; S.E.M.; *P<0.05). (**d**) A titration of E/T ratio in the redirected killing assay was performed. Shown is the percent target cell killing by WF-10 (200 *μ*M) for the respective E/T ratio (each value compared with w/o WF-10). The figure shows one out of two experiments with similar results.

Since GrA polarization into the cytolytic immune synapse and degranulation was intact, we hypothesized that WF-10 could alter the stability of E/T couples. We, therefore, analyzed the percentage of E/T couples by InFlow microscopy 15 and 75 min after mixing effector and target cells (Figure 3a). As expected, a significantly increased number of CTLs bound to OKT-3-bearing target cells as compared with unloaded target cells after 15 min. The numbers of E/T couples further increased when CTLs were pretreated with WF-10. While numbers of E/T couples decreased after 75 min under control conditions (compare white bars in Figure 3a), WF-10-treated CTLs showed no decrease in the amount of E/T couples over time (compare black bars in Figure 3a) suggesting a stabilization of existing cell couples.

Stabilized E/T couple formation may increase CTL-mediated killing of a single target cell. However, the ability of CTLs to kill multiple target cells might be affected. To further delineate the impact of WF-10 on CTL-mediated serial killing we performed time-lapse video microscopy (TLV). In these experiments, we limited the ratios between effector and target cells in a way that CTLs had no multiple target cell contacts at the beginning of the experiment. In control conditions, target cells showed the first signs of apoptosis (rounding, shrinking and bleb formation) 15 min after the first contact with CTLs. CTLs were able to move from one apoptotic target cell to another target cell and to induce apoptosis in this second target cell (Figure 3b, upper row and Supplementary Movie S1). Disengagement from target cells was facilitated by target cell death, which is in line with earlier reports showing that detachment of NK cells from target cells is dependent on caspase activity and apoptosis of target cells.^29^ As observed for control cells, WF-10-treated CTLs were able to kill the target cell. The kinetics of the induction of apoptosis was comparable to that observed for untreated CTLs. However, we found that pretreatment of CTLs with WF-10 inhibited their attachment to a second target cell, even if a clear contact to the second target cell occurred (Figure 3b, lower row and Supplementary Movie S2). The quantification in Figure 3c demonstrates that in the presence of WF-10 significantly less CTLs show serial killing, that is, kill more than one target cell.

An increase in the E/T ratio should overcome this inhibitory effect, if the inhibition of target cell killing by WF-10 was due to a mere defective serial killing. We therefore titrated the E/T ratio from 3/1 to 24/1 and measured the influence of WF-10 on target cell killing for each condition (Figure 3d). We found a linear increase of killing efficiency from ratios 3/1 to 12/1 and a strong increase, if a ratio of 24/1 was used. This result is clearly in line with the finding that serial killing and not the killing machinery by itself was affected, since an increase in CTL number makes serial killing unnecessary and thus renders CTLs refractory towards WF-10.

### Increased LFA-1 enrichment in the E/T immune synapse after WF-10 treatment

**Figure 4:**
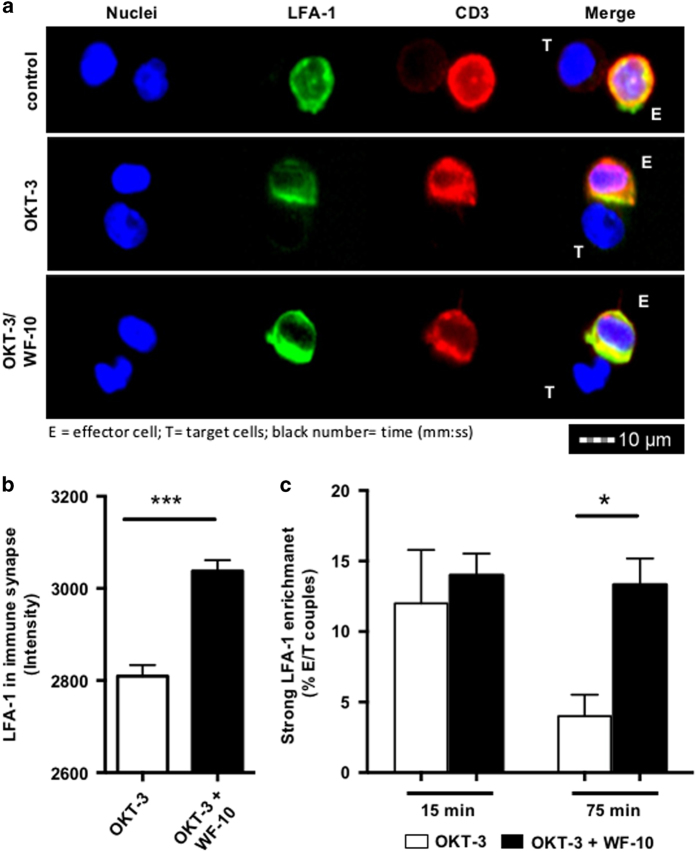
Enhanced LFA-1 enrichment in the cytolytic immune synapse in WF-10-treated CTLs. (**a**) InFlow microscopy images show representative E/T couples from redirected killing assays. The nuclei were stained with Dapi (blue), LFA-1 with anti-CD18-FITC antibodies (green) and CD3 with anti-CD3-PeTxR antibodies (red). The merge shows the overlay of all colors. The images are representative for four independent experiments. Yellow indicates the overlay of red and green. (**b**) The fluorescence intensity of LFA-1 was calculated in the E/T contact zone (=cytolytic immune synapse) (*n*=3; S.E.M.; ***P<0.001). (**c**) CTLs of E/T couples that had a strong LFA-1 enrichment, that is, at least 50% of the cellular LFA-1, were quantified for control or WF-10-treated CTLs and for two time points (15 and 75 min) (*n*=3; S.E.M.; *P<0.05).

We next sought to investigate the underlying reason for sustained cytolytic synapse formation and eventual inhibition of serial killing. One possible reason for the inhibition of serial killing could be an increased adhesion of CTLs to their (initial) target cells. An important adhesion molecule that mediates such cell/cell interactions is LFA-1.^30^ We therefore measured LFA-1 clustering at the E/T immune synapse using InFlow microscopy (Figure 4a). We found that LFA-1 was equally distributed on the CTL surface in the absence of OKT-3. LFA-1 enriched in the cytolytic immune synapse in the presence of OKT-3. This strong LFA-1 enrichment was transient, as the number of cells with a strong LFA-1 enrichment in the cytolytic immune synapse reduced over time (Figure 4c). In contrast to control conditions, WF-10 pretreatment of CTLs led to a profound (Figure 4b) and sustained (Figure 4c) increment of LFA-1 enrichment in the cytolytic immune synapse. Such an increase in LFA-1 concentration in the cytolytic immune synapse provides a molecular explanation for the inhibition of serial killing by WF-10.

### F-actin increment and hyper-phosphorylation of L-plastin by WF-10

**Figure 5:**
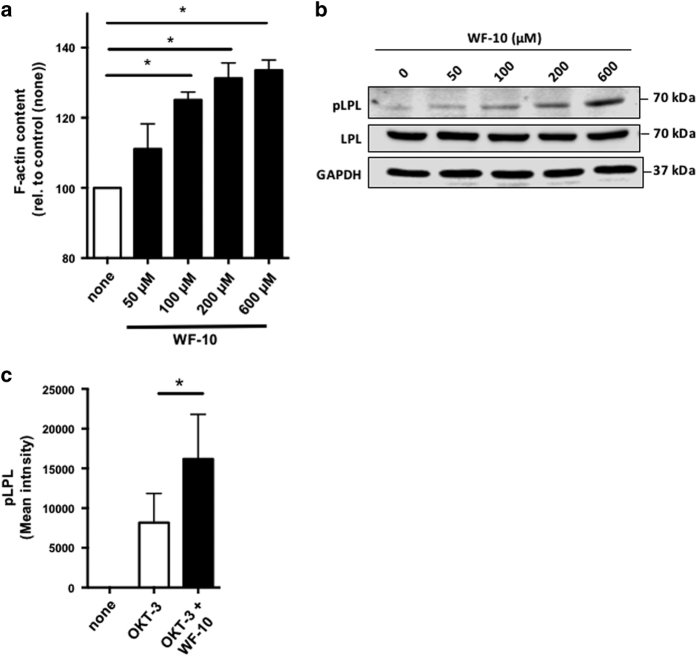
WF-10 induced L-plastin phosphorylation. (**a**) F-actin content in CTLs was analyzed by flow cytometry. CTLs were incubated with WF-10 for 2 h and stained with SiR actin (*n*=3; S.E.M.; *P<0.05). (**b**) CTLs were incubated with WF-10 for 2 h as indicated. After cell lysis, cytoplasmic fractions were prepared and proteins were subjected to western blot analysis using antibodies that are specific for phopsho-L-plastin (pLPL), L-plastin (LPL) or GAPDH. The blot is representative for three independent experiments. (**c**) Phosphorylation of L-plastin was analyzed in E/T couples using InFlow microscopy. The time point is 75 min after E/T couple formation (*n*=3; S.E.M.; *P<0.05).

Enhancement of avidity of LFA-1 in the immune synapse between T-cells and APCs is dependent on the actin cytoskeleton.^4^ The higher LFA-1 enrichment in the cytolytic immune synapse could, therefore, be due to a missregulated actin cytoskeleton. In line with this assumption, we found a concentration-dependent increase of the F-actin content in CTLs that were treated with, respectively, 50, 100, 200 or 600 *μ*M WF-10 for 2 h as measured by flow cytometry (Figure 5a).

An important factor for F-actin stabilization and actin-dependent LFA-1 regulation is L-plastin.^4^ The activity of L-plastin is positively regulated by phosphorylation on Ser5, for example, upon T-cell costimulation.^3^ We therefore analyzed the phosphorylation state of L-plastin following WF-10 treatment of CTLs by western blot. WF-10 induced phosphorylation of L-plastin in resting CTLs in a dose-dependent manner (Figure 5b). We next analyzed the phospho-L-plastin content in CTLs of E/T couples using InFlow microscopy (Figure 5c). Under control conditions, L-plastin phosphorylation could only be observed in the presence of OKT-3, suggesting that L-plastin activity is important to enable CTLs to kill target cells. Interestingly, the amount of phospho-L-plastin was significantly higher in WF-10-pretreated cells.

**Figure 6:**
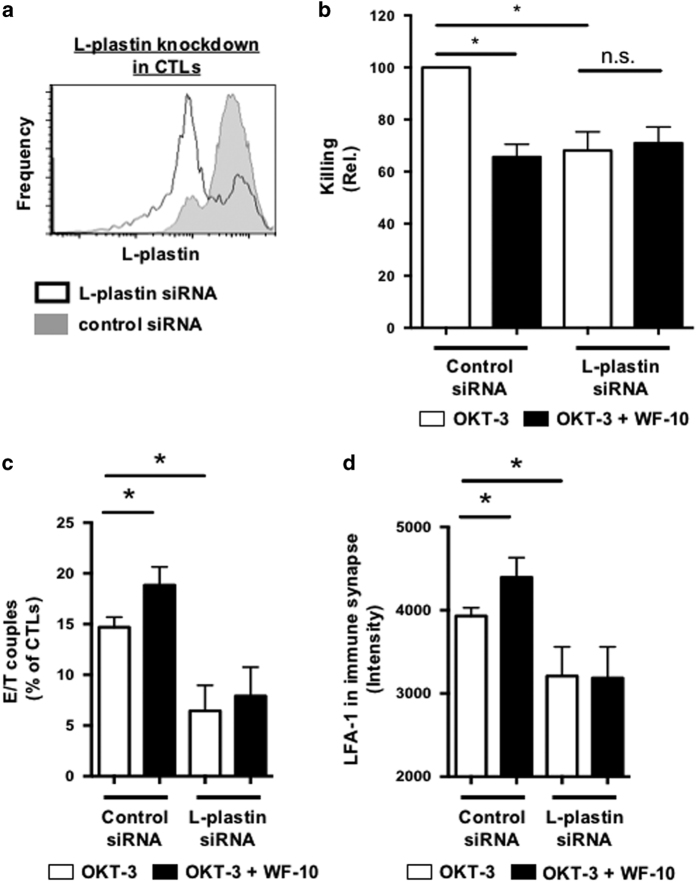
L-plastin is important for CTL-mediated target cell killing. (**a**) The InFlow microscopy histogram shows downregulation of L-plastin expression by L-plastin-specific siRNA. L-plastin was stained with polyclonal antibodies and secondary detection with PE-labeled antibodies. (**b**) Target cell killing by control siRNA or L-plastin siRNA-treated cells in the presence or absence of WF-10 was measured by InFlow microscopy. Shown is the target cell killing relative to control siRNA-treated cells in the absence of WF-10 (*n*=3; S.E.M.; *P<0.05). (**c** and **d**) Percent E/T couples (**e**) as well as LFA-1 amount in the cytolytic immune synapse (**f**) of CTLs, which were either treated with control siRNA or with L-plastin-specific siRNA, was measured using InFlow microscopy as described above (*n*=3; S.E.M.; *P<0.05). White bars: absence of WF-10; black bars: presence of 200 *μ*M WF-10

To examine the function of L-plastin for CTL-mediated target cell killing, we generated CTLs lacking L-plastin using siRNA (Figure 6a). In line with the finding that L-plastin undergoes phosphorylation in CTLs upon stimulation via OKT-3-bearing target cells, we found that L-plastin is important for CTL-mediated killing, since a knockdown of L-plastin in CTLs significantly reduced the amount of apoptotic target cells (Figure 6b, compare white columns). The degree of inhibition was comparable to that observed for WF-10 and control siRNA treated CTLs. Notably, preincubation of L-plastin knockdown cells with WF-10 did not further decrease the ability of CTLs to kill their target cells, implying that WF-10 may exert its effects via L-plastin. Presumably, the inhibition of CTL-mediated killing by the L-plastin knockdown is due to unstable initial E/T cell couples since L-plastin is important for cell/cell contacts.^4^ Consistent with this assumption, the number of E/T couples was significantly reduced in L-plastin knockdown cells compared with control siRNA-treated cells (Figure 6c, white bars). WF-10 led to a significant rise of the percentage of E/T couples in control cells, but not in L-plastin knockdown T-cells. In parallel, the amount of LFA-1 in the cytolytic immune synapse was significantly reduced in L-plastin knockdown T-cells (Figure 6d, black bars). While preincubation of CTLs with WF-10 alone led to a rise of LFA-1 in the cytolytic immune synapse, it did not increase the respective amount of LFA-1 in L-plastin knockdown T-cells. Thus, this finding supports the notion that L-plastin is critically linked to activities induced by WF-10 in CTLs.

### Additive effects of WF-10 and FK506 or CsA on inhibition of CTL-mediated killing

**Figure 7:**
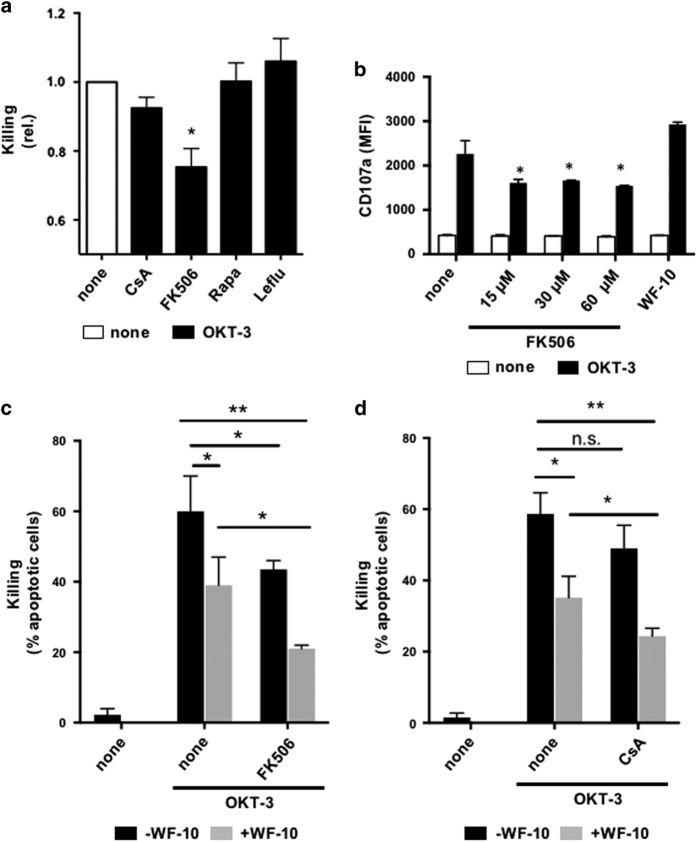
FK506 or CsA in combination with WF-10 have additive inhibitory effects on CTL-mediated target cell killing. (**a**) CTL-mediated killing was measured after preincubation with immunosuppressive drugs as indicated. Each value was normalized to control CTLs stimulated in the absence of drugs (none) (*n*=3; S.E.M.; *P<0.05). (**b**) Degranulation of CTLs that were either incubated with FK506 (concentrations as indicated) or 200 *μ*M WF-10 was measured by CD107a expression level (MFI) using flow cytometry (*n*=3; S.E.M.; *P<0.05). (**c** and **d**) CTLs were either preincubated with solvent control (none) or 30 nM FK506 (**c**) or 1 *μ*g/ml CsA (**d**) either in the absence (black bars) or presence of 200 *μ*M WF-10 (gray bars). Thereafter, target cell killing was assessed by measuring apoptotic target cells using InFlow microscopy (*n*=3; S.E.M.; NS, not significant; *P<0.05; **P<0.01).

Given the specific effect of WF-10 on T-cell/target cell adhesiveness, but not degranulation, we next asked if WF-10 could complement effects of clinically used immunosuppressive drugs. To get an overview about the inhibitory effects of clinically used immunosuppressive drugs on CTL-mediated killing, we preincubated CTLs with cyclosporine A (CsA; 1 *μ*g/ml), FK506 (30 nM), rapamycin (Rapa, 100 nM) or leflunomide (Leflu, 100 *μ*M) for 30 min. Of those, FK506 showed the strongest inhibition on CTL-mediated killing in the tested concentration (Figure 7a). Note that in the tested concentrations all drugs were able to inhibit T-cell activation without being toxic (Supplementary Figure S3).

Since FK506 showed the strongest inhibitory effect, we tested this calcineurin inhibitor in more detail. In contrast to WF-10, FK506 inhibited degranulation of CTLs as measured by CD107a surface expression (Figure 7b). This shows that the molecular mechanism by which this drug inhibits target cell killing differs from that observed for WF-10. Notably, an increase of FK506 concentration did not lead to a stronger suppression of degranulation, which shows that a higher dose of this drug during treatment of patients would presumably not improve CTL suppression. Given the mechanistic differences of WF-10 *versus* FK506 mediated inhibition in target cell killing and the saturation of FK506 effects on CTL-mediated killing, it was tempting to speculate that a combination of both drugs could synergize and, thus, increase the cytotoxic effect compared to single drug usage. We, therefore, next preincubated CTLs with FK506 alone or in combination with WF-10 and measured target cell killing (Figure 7c). Indeed, we found that WF-10 has an additive effect on the inhibition of target cell killing by FK506.

CsA, another calcineurin inhibitor, showed only a statistically nonsignificant inhibitory trend on CTL-mediated killing (Figures 7a and d). Higher CsA concentrations did not further increase the inhibition of CTL functions (data not shown). Interestingly, a combination of CsA and WF-10 displayed a similar synergistic inhibitory effect on CTL-mediated target cell killing as obtained by a combination of FK506 and WF-10 (Figure 7d). Altogether, WF-10 could complement the immunosuppressive functions of calcineurin inhibitors by enabling effective inhibition of cytotoxic activity of T-cells.

## Discussion

T-cell mediated cytotoxicity is required to eliminate foreign invaders (particularly viruses) or cancer cells. However, cytotoxic T-cells can also distruct healthy tissues, which is detrimental during autoimmune disorders, as, for example, type 1 diabetes (T1D). Therefore, the functionality of cytotoxic T-cells needs to be tightly controlled. The tissue micromilieu is a key determinant for the function of cytotoxic T-cells.^31^ Among the many immunomodulating factors of the microenvironment, critical factors include cytokines, metabolites and redox-active substances. Here, we investigated whether the redox-active compound WF-10 modulates killing by activated effector CD8^+^ T-cells, that is, CTLs. We found that WF-10 significantly impaired serial killing of target cells. Earlier events underlying target cell killing, that is, GrA polarization and degranulation, were not affected by WF-10.

The inhibition of serial killing by WF-10 was accompanied by a stronger enrichment of LFA-1 in the cytolytic immune synapse. LFA-1 is a major mediator for adhesion of T-cells to other cells^30,32^ and CTL-mediated killing.^33^ A firm adhesion of CTLs to their target cells is important for the formation of a cytolytic immune synapse and to induce apoptosis of target cells. We show here that under normal conditions (in the absence of WF-10) an initial increase in synaptic LFA-1 is followed by a time-dependent decrease. While the initial LFA-1 recruitment to the cytolytic immune synapse is crucial for killing of the first target cell, the time-dependent reduction of LFA-1 in the cytolytic immune synapse allows uncoupling from the first target cell and attachment to another target cell – eventually leading to serial killing. Therefore, LFA-1 affinity and avidity need to be temporally regulated in CTLs. Interestingly, WF-10 treatment resulted in enhanced and prolonged LFA-1 clustering in the cytolytic immune synapse and led to elevated E/T couple formation. Despite this increase in adhesiveness and couple formation, CTL-mediated killing was significantly inhibited by WF-10. A mechanistic explanation for this finding is that the enhanced and prolonged enrichment of LFA-1 in the cytolytic immune synapse in the presence of WF-10 stabilizes the T-cell contact to the first target cell and prolongs cell couple dwell times, which is adverse for serial killing of additional target cells.

The actin cytoskeleton is crucially involved in the regulation of LFA-1 avidity and formation of large LFA-1 clusters in the immune synapse. A constant actin flow regulates LFA-1 cluster formation in the cytolytic immune synapse.^34–39^ LFA-1 is intracellularly anchored to the actin cytoskeleton via talin^40^ and L-plastin.^4^ We have shown earlier that knockdown of the actin-bundling protein L-plastin in naive T-cells produces a disturbed LFA-1 enrichment in the early immune synapse between T-cells and APCs.^4^ The T-cell/APC dwell time dropped drastically as a consequence of this L-plastin knockdown and activation of naïve T-cells was prevented. Here we show that L-plastin is phosphorylated (more active form) in CTLs upon target cell engagement. This activation of L-plastin is crucial for CTL functionality, since a knockdown of L-plastin reduced both the number of E/T couples and CTL-mediated target cell killing. Interestingly, in the presence of WF-10 L-plastin appeared in its more active phosphorylated form, which may explain the diminished detachment of CTLs from their first target cell resulting in impaired serial killing. The assumption that the inhibitory effect of WF-10 on serial killing is at least in part mediated by WF-10-dependent phosphorylation of L-plastin is further substantiated by the finding that WF-10 had no influence on target cell killing in L-plastin knockdown CTLs.

Our findings have potential clinical implications, since WF-10 efficiently inhibits the serial killing capacity of CTLs, which is crucially involved in the progression of certain autoinflammatory diseases and transplant rejection. In line with this hypothesis graft survival in a concordant xenograft model was significantly prolonged in the presence of WF-10.^41–43^ Importantly, given that WF-10 has inhibitory effects on the late phase of T-cell activation, it also seems especially beneficial for patients with chronic inflammatory diseases. One example is T1D, in which CD8^+^ T-cell-mediated killing is involved in the pathogenesis (reviewed in Gravano and Hoyer,^11^ Santamaria,^12,13^ Walter and Santamaria,^14^ and Liblau *et al*.^15^) contributing to the clinical development of diabetic foot ulcer (DFU). Indeed, it was demonstrated that WF-10 improves the clinical outcome of DFU.^17^ In addition, we found that the clinically used immunosuppressive drug FK506 inhibits CTL-mediated killing by a different molecular mechanism, namely counteracting degranulation, which is also in line with earlier results.^44^ Thus, given that relatively high doses of FK506 are needed to inhibit CD8 T-cell functions the synergistic inhibitory effect of WF-10 and FK506 on CTL-mediated killing may provide an opportunity to block cytotoxicity more efficiently and to minimize side effects of each drug by adapting the respective concentrations. Even more important seems the finding that CsA alone has only marginal effects on CTL-mediated cytotoxicity, but it synergizes with WF-10 in target cell killing. Thus, WF-10 may efficiently complement classical immunosuppressive regimes to control progression of chronic inflammatory and autoimmune diseases as well as to prevent rejection of transplanted organs, in which cytotoxic T-cells are crucially involved. In addition, since WF-10 has no inhibitory effect on innate immune responses,^18^ WF-10 might provide the advantage of securing a residual protection against opportunistic infections, which is a common problem during immunosuppression.

## Materials and Methods

### Cell culture and reagents

The target cell line P815 was split every other day (1:25 in RPMI1640/10% FCS). T-cells were isolated using the pan T-cell isolation kit or CD8^+^ T-cell isolation kit, respectively, from Miltenyi Biotec (Bergisch Gladbach, Germany) as described elsewhere.^8^ CD8^+^ T-cells were stimulated with phytohemagglutinin (PHA-L, 2 *μ*g/ml) in cell culture medium (RPMI1640/10% FCS) at 37 °C for 24 h. After removal of PHA-L, cells were cultured with recombinant interleukin-2 (10 U/ml) at 37 °C for 7 days. The knockdown of L-plastin is described elsewhere.^4^ Briefly, an L-plastin specific or non-targeting control siRNA was transfected into CTLs using an nucleofector (Lonza, Basel Switzerland). Experiments were performed 5 days after transfection. To costimulate T-cells, antibodies against CD3 (5ng/ml) and CD28 (5 *μ*g/ml) were immobilized in 96-well plates. T-cells (2.5×10^5^/well) were spun down on the antibodies and incubated at 37 °C for 2 h. Then brefeldin A (end concentration 10 *μ*g/ml; purchased from Sigma-Aldrich, Taufkirchen, Germany) was added for another 4 h. After fixation (1.5% paraformaldehyde for 10 min) cells were stained with fluorescent antibodies against IL-2 and CD69 and the amount of IL-2 and CD69-expressing cells was analyzed using flow cytometry (for more details see Wabnitz *et al.*^3^). For testing cell viability after costimulation, cells were stained with 2.5 *μ*g/ml propidium iodide. Living cells (PI-negative) were detected using an LSRII (BD Bioscience, Heidelberg, Germany).

FK506, cyclosporin A, leflunomide as well as rapamycin were obtained from Sigma-Aldrich. WF-10 was kindly provided by Nuvo Research Inc. (Mississauga, ON, Canada). Brefeldin A, PHA-L and propidium iodide were bought from Sigma-Aldrich and IL-2 was purchased from Peprotech (Hamburg, Germany). All fluorescently labeled antibodies were bought from BD Bioscience.

### Killing assay (flow cytometry, InFlow microscopy and time-lapse microscopy)

The mastocytoma cell line P815 was used as target cell. For flow cytometry-based killing assays, these cells were labeled with CFDA (0.5 *μ*M CFDA for 0.5×10^6^ cells/ml) and adjusted to a cell density of 4×10^6^/ml (RPMI+2% FCS). To stimulate CTLs, P815 were loaded with anti-CD3 antibodies by incubation with 5ng/ml OKT-3 at 37 °C for 75 min. Note that loading of an isotype control antibody on P815 did not induce T-cell mediated killing or CD107a upregulation on killer cells. CTLs (4×10^6^/ml, RPMI+2%FCS) were incubated with the drugs (as indicated in the respective experiment) or solvent control (DMSO for immunosuppressive drugs or PBS for WF-10) for 2 h (WF-10 or H_2_O_2_) or 30 min (immunosuppressive drugs). Thereafter, CTLs and target cells were mixed at a ratio of 6:1 (or titrated as indicated). CD107a was trapped at the cell surface by adding 2.5 *μ*l APC-labeled anti-CD107a antibodies (BD Bioscience). Cells were stained with Annexin-V-PE before fixation and with CD3-PerCP after fixation. Acquisition of data was performed with an LSR2 and data were analyzed with FlowJo (FlowJo LLC, Ashland, OR, USA). CTLs and target cells were distinguished according to their CFDA-labeling and CD3 expression. Apoptotic target cells were identified by Annexin-V binding to CFDA^+^ cells and degranulation was measured by CD107a surface expression on CD3^+^ cells.

For InFlow microscopy experiments effector and target cells were mixed (6:1) and incubated for 15 or 75 min at 37 °C. Then, cells were fixed (1.5% paraformaldehyde), stained for nuclei (Dapi) and with antibodies against CD3 (PeTxR) and GrA (FITC) or LFA-1 (CD18-FITC). Images of 15 000 cells were acquired using an IS100 (Amnis Corp., Seattle, WA, USA) and evaluated using the software IDEAS version 6.0 (Amnis Corp.). CTLs and target cells were distinguished according to CD3 expression on CTLs and the sidescatter profile of target cells. Apoptotic cells were identified by evaluation of the nuclei. Defragmented nuclei have a smaller area and an increased 'bright detail intensity'. The latter is a feature that can be calculated by IDEAS software (for details see George *et al.*^45^). E/T couples were defined by combining the aspect ratio feature for nuclei and the sidescatter. GrA and LFA-1 polarization was analyzed by calculating a ratio of the GrA-derived fluorescence intensity signals in regions of interest that define the CTL and the cytolytic immune synapse (for details of cell couple analysis by InFlow microscopy see Wabnitz *et al*.^46^).

TLV microscopy was performed using a Nikon A1R (×60 objective; WI, NA 1.27; zoom 2.0; dimension 2253×2253 pixel). To this end, effector and target cells were placed on 35 mm dishes (Ibidi, Martinsried, Germany) at a ratio of 6 : 1 and a total amount of 1.8×10^5^ target cells and 2.5×10^5^ effector cells per dish. The experiment was conducted under full environmental control (37 °C and 5% CO_2_). Large brightfield images (5×5 optical fields) were acquired for 2 h with a 2 min time interval. E/T couple formation was analyzed by blinded optical evaluation of at least 50 cell couples for each condition. The experiment was performed four times with similar results.

### F-actin measurement

CTLs were cultured in the presence of indicated concentrations of WF-10 in RPMI1640 medium supplemented with 2% FCS at 37 °C, 5% CO_2_ for 2 h. Thereafter, 100 nM SiR actin (Spirochrome, Stein am Rhein, Switzerland) was added and cells were cultured for an additional hour. Samples were immediately measured by flow cytometry (LSRII).

### Phospho-L-plastin detection by western blot

1×10^6^ WF-10-treated cells were washed twice using ice-cold PBS and lysed using TKM lysis buffer (50 mM Tris-HCl pH 7.5, 1% NP40, 25 mM KCl, 5 mM MgCl_2_, 1 mM NaVO_4_, 5 mM NaF, 1% protease inhibitor cocktail (Sigma-Aldrich)) for 30 min on ice. Nuclei and cytosolic fragments were separated by 10 min centrifugation at 10 000 *g* at 4 °C. Cell lysates were separated by SDS-PAGE and electroblotted onto polyvinylidene difluoride membranes (PVDF; Immobilon-P, Merck-Millipore, Darmstadt, Germany). For detection of pLPL, membranes were incubated with pLPL antibody (own laboratory^47^) followed by IR680-conjugated secondary antibody (goat anti-rabbit, 1:10 000). For detection of LPL, membranes were incubated with LPL4A.1 (MA5-11921; Thermofischer, Darmstadt, Germany, 1:500) followed by IR800 conjugated secondary antibody (goat anti mouse IR800, 1:10 000)). The infrared signal was detected by a Licor Odyssey scanner (Bad Homburg, Germany). L-plastin phosphorylation was calculated as the ratio between phospho protein and total protein signal.

### Intracellular ROS detection

Cellular levels of ROS were determined using the redox sensitive probe CM-H_2_DCFDA (5- (and 6-) chloromethyl-29,79-dichlorodihydrofluorescein diacetate, acetyl ester) (Thermo Scientific, Darmstadt, Germany) according to the manufacturer´s instructions. Briefly, cells were washed once with PBS and incubated in 5 *μ*M CM-H_2_DCFDA in PBS at 37 °C for 10 min. Thereafter, cells were washed by centrifugation and immediately treated with the indicated concentrations of WF-10 and H_2_O_2_ for 5, 15 and 30 min in cell culture conditions. The oxidation of the probe was detected by an increase in fluorescence intensity using flow cytometry (LSRII).

### Statistics

The statistical analysis was performed with GraphPad Prism version 6.00 (STATCON, Witzenhausen, Germany) using *t*-test or paired *t*-test for matched observation.

## Notes

SM owns stock of Nuvo Research Inc. and has served as a scientific advisor until December 2014.
