# Supplementary material for: The pro-oxidative drug WF-10 inhibits serial killing by primary human cytotoxic T-cells
Source: Cell Death Discov. 2016 Jul 25;2:16057–. doi: 10.1038/cddiscovery.2016.57 (PMC4979520; doi:10.1038/cddiscovery.2016.57)
Supplement: Supplementary Figure [file cddiscovery201657-s1.pdf]

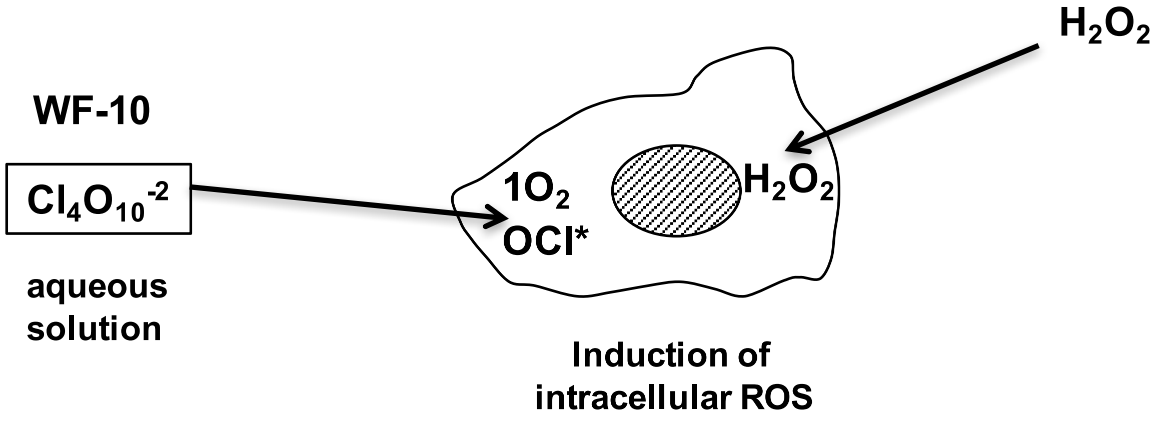


**Supplement Figure 1**
**WF-10 induces ROS in human CTLs**
A) WF-10 is a chlorite containing drug (1:10 dilution of tetrachlorodecaoxide), which is stable in aqueous solutions. It is converted by a Michealis-Menten reaction leading to production of ROS within the cytosol of cells. $H_2O_2$ also induces a pro-oxidative milieu in the cytoplasm without being processed.

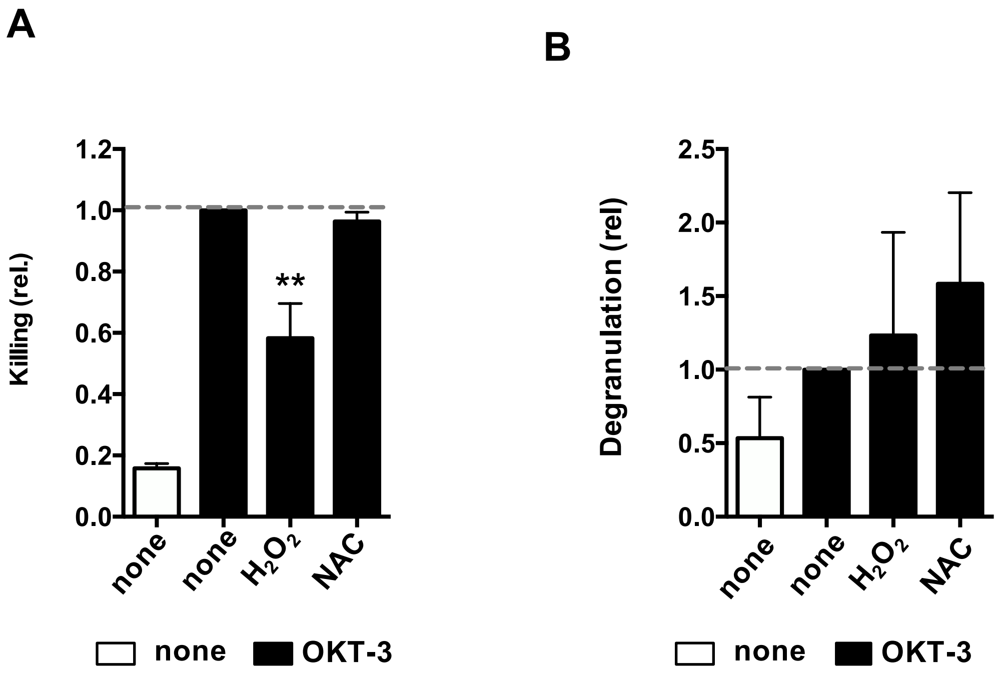


**Supplement Figure 2**

**$H_2O_2$ interferes with target cell killing by human CTLs**

A) Target cell killing was analyzed after mixing of CTLs with OKT-3-bearing (black bars) or uncoupled target cells (white bar for three independent experiments. CTL were preincubated with 50µM $H_2O_2$, 1mM NAC or solvent control (none) (n=3; SEM; *p<0.05).

B) Degranulation was analyzed in the same samples as described in A (n=3; SEM).

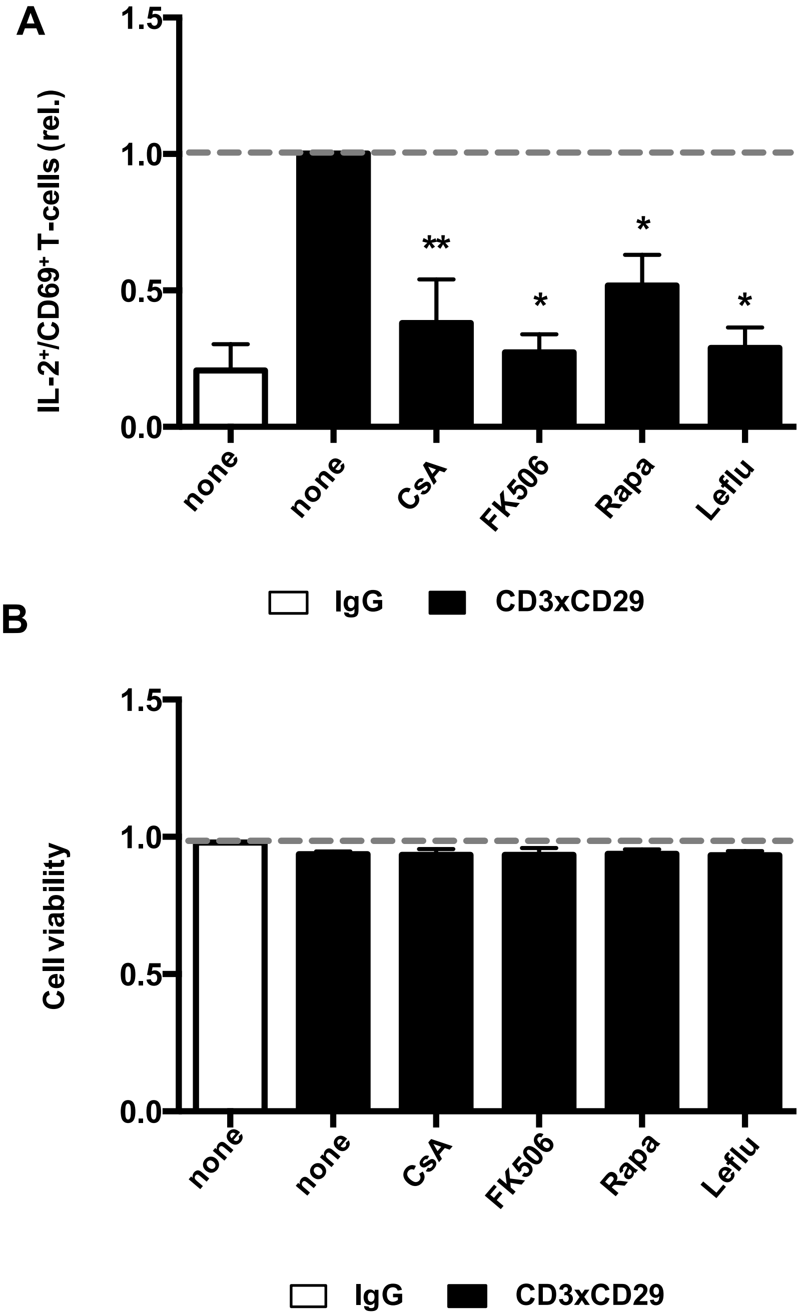


**Supplement Figure 3**

**Inhibition of T-cell activation by immunosuppressive drugs without toxic effects**

A) In the presence or absence of immunosuppressive drugs, as indicated, T-cells were stimulated via crosslinked antibodies against CD3 and CD28 (CD3xCD28, black bars) or settled on isotype control antibodies (IgG, white bars). T-cell activation was determined according to expression of IL-2 and CD69 measured by flow cytometry. Shown is the relative amount of activated T-cells (IL-2+/CD69+) (n=3; SEM; $^*p<0.05$; $^{**}p<0.01$).

B) The amounts of living T-cells (PI-negative) that were pre-incubated with immunosuppressive drugs and costimulated (CD3xCD28) for three days are depicted (black bars). The amount of living cells that were settled on isotype control antibodies (IgG) is shown as white bar (n=3, SEM).
