# Supplementary material for: The pro-oxidative drug WF-10 inhibits serial killing by primary human cytotoxic T-cells
Source: Cell Death Discov. 2016 Jul 25;2:16057–. doi: 10.1038/cddiscovery.2016.57 (PMC4979520; doi:10.1038/cddiscovery.2016.57)
Supplement: Supplementary Information [file cddiscovery201657-s2.pdf]

Supplemental information

Timelapse videomicroscopy

The pro-oxidative drug WF-10 inhibits serial killing by primary human cytotoxic T-cells

Guido H. Wabnitz, Emre Balta, Silke Schindler, Henning Kirchgessner, Beate Jahraus, Stefan Meuer, Yvonne Samstag

Supplement Movie 1:

Timelapse movie of serial killing by CTLs

CTLs were mixed with target cells (P815) that were loaded with OKT-3. Bright field images were taken with a time interval of 1 minute. The CTL is labeled with an “E” (=effector cells) and target cells with a “T” in the first frames of the time lapse movie.

Supplement Movie 2:

WF-10 inhibits serial killing by CTLs

CTLs were pre-incubated with 200µM WF-10 and then mixed with OKT-3 loaded target cells (P815). Image acquisition and labeling was performed as described in supplement Movie. 1. Movie images are also shown in Fig. 3B of the manuscript.
